# Sociedad, comunicación y salud: Transitar la pandemia de covid-19 y después

**DOI:** 10.18294/sc.2024.5059

**Published:** 2024-06-08

**Authors:** Paula G. Rodríguez Zoya, Mónica Petracci, Alejandro Kaufman

**Affiliations:** 1 Doctora en Ciencias Sociales. Investigadora adjunta, Consejo Nacional de Investigaciones Científicas y Técnicas, con sede en Instituto de Investigaciones Gino Germani, Facultad de Ciencias Sociales, Universidad de Buenos Aires, Ciudad Autónoma de Buenos Aires, Argentina. paula.rzoya@gmail.com Universidad de Buenos Aires Instituto de Investigaciones Gino Germani Facultad de Ciencias Sociales Universidad de Buenos Aires Ciudad Autónoma de Buenos Aires Argentina paula.rzoya@gmail.com; 2 Doctora en Ciencias Sociales. Investigadora, Instituto de Investigaciones Gino Germani, Facultad de Ciencias Sociales, Universidad de Buenos Aires, Ciudad Autónoma de Buenos Aires, Argentina. mnpetracci@gmail.com Universidad de Buenos Aires Instituto de Investigaciones Gino Germani Facultad de Ciencias Sociales Universidad de Buenos Aires Ciudad Autónoma de Buenos Aires Argentina mnpetracci@gmail.com; 3 Profesor e Investigador, Instituto de Investigaciones Gino Germani, Facultad de Ciencias Sociales, Universidad de Buenos Aires; Centro de Estudios en Historia, Cultura y Memoria, Universidad Nacional de Quilmes, Ciudad Autónoma de Buenos Aires, Argentina. alekau@gmail.com Universidad Nacional de Quilmes Centro de Estudios en Historia, Cultura y Memoria Universidad Nacional de Quilmes Ciudad Autónoma de Buenos Aires Argentina alekau@gmail.com; Instituto de Investigaciones Gino Germani Facultad de Ciencias Sociales Universidad de Buenos Aires Ciudad Autónoma de Buenos Aires Argentina

## PRESENTACIÓN

Esta convocatoria de *Salud Colectiva* surgió en el marco del desafío global que representó la pandemia de covid-19, así declarada por la Organización Mundial de la Salud el 11 de marzo de 2020 ante el crecimiento del número de casos y de países afectados fuera de China (país en el que, en la ciudad de Wuhan, se produjeron las primeras notificaciones en diciembre del año anterior).

La complejidad, el carácter global y la magnitud de la pandemia exteriorizan, en su dimensión de época, los retos de las relaciones entre sociedad, comunicación y salud. La irrupción de la pandemia modificó -puso “en pausa”- la cotidianeidad planetaria y los discursos constitutivos y constituyentes de las subjetividades contemporáneas. Alteró los modos de relacionarnos, la habitualidad del rostro visible, el aislamiento y el distanciamiento de los cuerpos, los modos de trabajo (en caso de mantenerlo), la educación, el cuidado y la atención de la salud; todo ello atravesado por los procesos de digitalización. La concepción del espacio y del tiempo, y la de la muerte, solo por citar algunos significantes ordenadores de las subjetividades, comenzaron brutalmente a interpelar la vida diaria, que devino supervivencia. Asimismo, la pandemia profundizó desigualdades sociales, económicas y de género, confrontaciones en el espacio público y polarizaciones políticas, brechas educativas e info-comunicacionales; así como también desplegó debates sobre el rol del Estado, las ciencias, el ambiente, el hábitat urbano, y las tecnologías de información y comunicación.

La pandemia de covid-19 fue un fenómeno que hizo estallar muchas de las coordenadas establecidas hasta ese momento y generó tantos interrogantes como incertidumbre ante un mundo que se veía amenazado y transformado en su orden, sus saberes, representaciones y prácticas por un virus cuyos efectos se volvían masivos. En esta clave, hablar de efectos, no remite solo al ámbito sanitario y epidemiológico, sino también al político-gubernamental, doméstico-habitacional, ciudadano, subjetivo y prácticamente a todos los planos de la existencia humana que fueron atravesados por la pandemia.

Así también el ámbito científico y académico fue sacudido e interpelado por esta crisis y se dio a una de las tareas que más lo representan: la de hacerse preguntas y buscar respuestas. Detrás de los interrogantes que la cuestión pandémica suscitó y continúa provocando, se desplegaron variados y vastos estudios en tiempo real que pusieron el foco en las circunstancias presentes que estaban siendo trastocadas. Las mismas prácticas y temas de investigación fueron reorganizados de cara a problematizar, conocer y comprender las condiciones de la pandemia y generar desarrollos para intervenir en ella. Fueron realizados desde sondeos psicosociales, relevamientos cuantitativos, etnografías, ensayos teóricos en ciencias sociales hasta estudios biológicos y médicos, tal como los emblemáticos -y no menos controversiales- desarrollos de vacunas contra el coronavirus en tiempo récord. La pandemia es un fenómeno multidimensional que ha trascendido las fronteras -bien delimitadas, aunque muchas veces cuestionadas- de los campos disciplinarios. La pandemia es un fenómeno multidisciplinar.

A partir de este marco general, queremos profundizar en algunos aspectos comunes a los temas y abordajes de los artículos publicados^1^^,^^2^^,^^3^^,^^4^^,^^5^^,^^6^^,^^7^^,^^8^: la pandemia como acontecimiento transicional; la pandemia como lente para problematizar relaciones de sociedad, comunicación y salud; y la concepción de la pandemia como fenómeno multidimensional y multidisciplinar.

### La pandemia como acontecimiento transicional

La noción de acontecimiento nombra un suceso por la novedad y magnitud de las alteraciones que provoca y sus consecuencias. La noción de transición también implica transformación, aunque en ello no hay un sentido claro del inicio o de los momentos del cambio al que se asiste; hay más bien un efecto de deslizamiento, un proceso que va consolidando modificaciones. Sostenemos que la pandemia de covid-19 conjuga ambos matices semánticos, el del acontecimiento y el de la transición; es un acontecimiento transicional cuyo efecto de estallido aún no ha dejado de resonar. La expresión del título que dimos a esta convocatoria, “Transitar la pandemia de covid-19 y después”, condensa esta idea e invita a pensar diversas huellas de ese tránsito.

Sin intención de periodizar ese tiempo podemos reconocer algunos rasgos y efectos de las circunstancias vividas. El año 2020 fue comúnmente experimentado con mayor incertidumbre por la novedad y el desconocimiento que implicó la eclosión de la pandemia. En 2021 fue común la percepción de que la extensión en el tiempo de esa coyuntura, la virtualización de diversas prácticas y actividades, y la consecuente sobreexposición tecnológica produjo agotamiento, malestares y sobrecarga en el desarrollo de las labores. La incertidumbre y restricciones establecidas para circular o incluso salir de los hogares generaron una clara dificultad para poder planificar actividades de la vida en general, lo que tiene un impacto en términos de representación de futuro. A raíz de los riesgos sociosanitarios se añadieron nuevas demandas en la gestión doméstica y laboral, tareas adicionales de higiene y cuidado de la salud, así como el sustento de redes familiares para el cuidado de personas mayores, niñas y niños y personas en grupos de riesgo. Todo ello conllevó transformaciones en el uso y representación del tiempo.

Por su dimensión global y por la velocidad de la diseminación epidemiológica y comunicacional con que se consumaron los acontecimientos, la pandemia introdujo la necesidad de repensar transitoriamente múltiples aspectos. Entre estos podemos mencionar, por ejemplo, las relaciones internacionales, tanto en lo institucional como en la producción de conocimiento científico y su difusión. Inédito fue el propósito hasta cierto punto alcanzado de disponer el libre acceso a la información científica habitualmente restringida por suscripción, por ejemplo, o las incidencias complejas de la validación y distribución de vacunas.

La vacunación, un tema central desde 2021, fue atravesada por la competencia entre países, corporaciones y organismos no gubernamentales por múltiples litigios y rivalidades, desde las validaciones científicas heterogéneas en calidad, reconocimiento y normas entre diversos países y regiones, la colisión entre intereses comerciales y de patentes, el acaparamiento de las vacunas por algunos países, los movimientos antivacunas, la politización ideológica, y la manifestación global y simultánea de las desigualdades intra e internacionales.

A partir de las vacunaciones masivas -atravesadas sin duda por fuertes debates e irregularidades en su distribución- ingresamos a un nuevo momento de la pandemia en el que se puso en juego la vuelta a la presencialidad y nuevas formas de cuidados y de negligencias. Asistimos a un paisaje transicional dentro del cual podemos distinguir los siguientes aspectos: 1) la instalación de una *nueva precariedad* vinculada con las incertidumbres sobre nuevas variantes emergentes de coronavirus, el manejo ambiental de los aerosoles y nuevas problemáticas edilicias y urbanas concernientes a la ventilación, la aparición de nuevas minorías segregadas de hecho por vulnerabilidades y comorbilidades desatendidas en el contexto de reanudación de la circulación social; 2) la vacancia de una conciencia social y de una cultura epidemiológica masiva y actualizada, interdisciplinaria y constitutiva de una ciudadanía informada; 3) la afectación de las institucionalidades estatales después de un lapso de entre un año y dos de incertidumbres y pérdida o deterioro de la gubernamentalidad; 4) la reputación y estatuto de los discursos científicos y médicos frente a las sociedades después del mismo lapso de exposición a una esfera pública en estado de pánico; 5) las condiciones de los miedos colectivos, la elaboración del duelo consecutivo a la calamidad y la repercusión traumática general suscitada por la pandemia, así como la constitución de subjetividades que condensan tales condiciones; 6) los medios de comunicación masivos como escenario de los acontecimientos pandémicos y las múltiples incidencias a que dieron lugar desde la crisis del periodismo hasta la reconfiguración de prácticas digitales.

La Organización Mundial de la Salud ha establecido el fin del covid-19 como emergencia sanitaria internacional el 5 de mayo de 2023, a más de tres años de haber declarado su inicio como pandemia. En 2024, las circunstancias son realmente otras, al punto de que el escenario pandémico se desdibuja y hasta parece ajeno o lejano. La pandemia ha iluminado falencias, errores, secuelas, nuevos saberes y necesidades a partir de los cuales es posible construir una agenda de trabajo que nos permita capitalizar los aprendizajes para que el tránsito por esa experiencia no haya sido en vano.

### La pandemia como lente para problematizar relaciones de sociedad, comunicación y salud

La pandemia delineó un complejo entramado de cuestiones que interpelan a las sociedades en términos comunicacionales y de salud. Esta afirmación no implica que la pandemia sea comprendida exclusivamente como un problema de comunicación y salud; hemos hecho referencia ya a la comprensión multidisciplinar de este fenómeno. Asimismo, lo que comprendemos por comunicación y por salud no es monolítico, interesan justamente los diversos modos en que estos términos se relacionan y se convierten -los construimos- en lentes de procesos sociales mayores y ángulos de observación de la pandemia.

Ejemplo de esta imbricación son, por ejemplo, las estrategias de comunicación pública de decisiones políticas en materia de salud; la información y divulgación periodística sobre la pandemia; la discursividad social, sentidos y representaciones relativos al virus, la pandemia, la cuarentena, el aislamiento y los riesgos de contagio; la promoción de nuevos hábitos de cuidado de la salud; las relaciones médico-paciente en la atención de salud; y las prácticas de salud digital mediadas por tecnologías de información y comunicación, que han tenido un gran impulso en ese contexto, entre otros temas posibles.

La pandemia generó cambios extraordinarios y la aceleración de diversos procesos sociales, epidemiológicos y técnicos. Una de las mayores transformaciones consistió en la virtualización de distintas prácticas sociales cuya presencialidad se vio impedida a raíz del riesgo de contagio y las medidas de cuarentena y aislamiento. La pandemia ha sido un gran propulsor de los procesos de digitalización que se extendieron a las prácticas laborales, educativas, comerciales y así también las de salud. La asistencia y gestión de la salud sufrió el impacto de las transformaciones digitales para adaptarse a las nuevas necesidades y circunstancias, dando lugar a un acelerado despegue de la telemedicina y al desarrollo de distintos tipos de dispositivos y aplicaciones móviles con usos específicos para el seguimiento de la vacunación, control de sintomatología y contagios. Esas nuevas plataformas informáticas en salud generaron también nuevos modos de comunicación.

El veloz desarrollo de la pandemia exigió además que los sistemas de salud sean adaptados para atender las nuevas demandas, ya sea por coronavirus o por otras afecciones o controles regulares de salud. La adecuación de la estructura y los recursos sanitarios a las nuevas condiciones exigidas por la pandemia incluyó la ampliación del número de camas de terapia intensiva, el montaje de hospitales de campaña, postas de vacunación, puestos de hisopado, compra de insumos médicos, entre otros. Esta reconversión sanitaria tiene su correlato en el ámbito científico con el desarrollo de los compuestos y las plataformas de las vacunas, y en el ámbito gubernamental, que requirió tanto del desarrollo de sistemas informáticos para la gestión de las políticas sanitarias como de estrategias comunicacionales para implementarlas y hacerlas públicas.

Cómo y desde qué perspectiva comunicar la pandemia fue uno de los tantos desafíos a los que funcionarios gubernamentales (desde presidentes hasta tomadores de decisión ministeriales) tuvieron que dar respuesta. Consideramos que la pandemia requirió, sobre todo, de los lineamientos de una comunicación de crisis por lo repentino, desconocido, la presencia de contagios y muertes, y la necesidad de respuestas urgentes.

Un debate experto puso frente a frente la pertinencia y los aportes de las comunicaciones de riesgo y de crisis en momentos de la pandemia. Para ejemplificar -tomando como base el caso argentino, y sin un derrotero evaluativo de lo realizado en nuestro país, en la región o en otras latitudes- podemos pensar (con la distancia temporal del tantas veces acudido “diario del lunes”) que si en febrero de 2020, cuando se empezaba a conocer qué estaba ocurriendo en China, se hubiese implementado un esquema comunicacional de riesgo, las situaciones de crisis sanitaria, como resultado de la insuficiencia o el desacierto de las medidas aplicadas, podrían haber sido más atenuadas por el inicio de la vigilancia epidemiológica, el conocimiento y la puesta en marcha de prácticas de prevención por la ciudadanía y los sistemas de atención.

Por muchas razones entre las que podemos resaltar la complejidad del escenario pandémico y las especificidades de cada país, la toma de decisiones conlleva solapamientos comunicacionales y no responde con prolijidad a un esquema secuencial de tipos de comunicación o a un debate planteado en términos binarios. Sencillamente porque quienes tienen que decidir e implementar una comunicación de riesgo desconocen el grado de riesgo a lo largo del proceso (aunque conozcan las consecuencias de sobrevalorarlo o subvalorarlo para las acciones gubernamentales, en medios de comunicación y redes sociales). Hasta la misma Organización Mundial de la Salud, que recomienda una comunicación planificada, con anuncios tempranos e información clara tuvo idas y vueltas sobre, por ejemplo, el uso de barbijos en los primeros momentos de la pandemia, cuando aún se desconocía la naturaleza de los aerosoles como factores decisivos de la propagación del agente infeccioso.

Lo anterior no significa, de ninguna manera, sucumbir en la incertidumbre, sino construir una praxis multidisciplinar que, de modo permanente, debata sobre modelos de atención y de comunicación en posibles escenarios pandémicos futuros. En ese sentido, en 2021 -dadas las consecuencias de la pandemia de covid-19- los 194 Estados miembros de la Organización Mundial de la Salud iniciaron un proceso mundial para redactar un convenio, acuerdo u otro tipo de instrumento internacional sobre preparación y respuesta frente a pandemias. De acuerdo al organismo internacional, los factores mencionados por los gobiernos para implementar una iniciativa de este tipo estuvieron centrados en la pérdida de vidas, las perturbaciones sufridas tanto en los hogares como en las sociedades, y las repercusiones posteriores a fin de garantizar que las comunidades, los gobiernos y todos los sectores de las sociedades estuvieran mejor preparados y protegidos ante futuras pandemias, garantizar la equidad en el acceso (en particular, a vacunas, equipos de protección personal, información y conocimientos especializados). Las conclusiones de las audiencias públicas serán presentadas y sometidas a la consideración de la 77ª Asamblea Mundial de la Salud, que se realizará en Ginebra, Suiza, del 27 de mayo al 1 de junio de 2024.

Otro aspecto que considerar, ya existente pero que respondió a las medidas gubernamentales de aislamiento social y preventivo, es de orden político-cultural. Marchas anti-cuarentena que cuestionaron desde la existencia misma del virus hasta la efectividad de las vacunas en consonancia con medios de comunicación hegemónicos cuyos principales ejes de discusión buscaron partir las aguas entre una criticada presencia del Estado y una aclamada libertad neoliberal. Dicho en otros términos: entre el cuidado de la ciudadanía y el mercado, atrapado siempre en una lógica dicotómica en el que ambos parecen excluirse mutuamente.

Los cambios acaecidos en términos de digitalización de las prácticas sociales y de salud, las experiencias en relación con estrategias de comunicación gubernamental, el tratamiento de la situación en medios masivos de comunicación, las iniciativas de parte de organismos internacionales para normalizar y uniformizar estrategias de acción ante futuros acontecimientos pandémicos, y la efervescencia de procesos comunicacionales en el espacio público que ponen de relieve la disputa por sentidos sociales sobre significantes ordenadores de una coyuntura crítica; todo ello son ejemplo del anudamiento de la comunicación y la salud como lente de problematización social, y así también marcan desafíos futuros para los países en la agenda que abrió el tránsito de la pandemia.

### La concepción de la pandemia como fenómeno multidimensional y multidisciplinar

Sobre la base de lo expuesto, adquiere todo su sentido reafirmar la concepción de la pandemia de covid-19 como un fenómeno multidimensional y un problema complejo que entrelaza el punto de vista de múltiples actores y requiere abordajes multidisciplinares, no restringidos a un único campo epistémico, especialidad o enfoque. En correspondencia con esta perspectiva, la convocatoria de artículos trazó un amplio arco temático a modo de disparadores para la reflexión crítica de la pandemia de covid-19, organizado en diez dimensiones.

- *Dimensión de la digitalización*: auge y expansión de prácticas virtuales y digitales en los ámbitos de la salud, el trabajo y la educación durante la pandemia. Implicancias de la virtualidad para la atención médica, el desarrollo de diversas prácticas de *eHealth* o salud digital, la organización del trabajo y la producción, las condiciones laborales y educativas, la producción y uso de datos en salud y *Big Data*, así como las implicancias subjetivas, éticas y políticas.
- *Dimensión comunicacional-informativa*: comunicación pública, gubernamental, mediática y periodística de la pandemia. Redes sociales, circulación de información y validación de fuentes informativas. Información, desinformación y contradiscursos. Controversias respecto a momentos y modos comunicativos de información sensible de la situación sanitaria y pautas de prevención.
- *Dimensión biopolítica*: políticas y debates en torno a regímenes de control de los cuerpos en pandemia, nuevas formas de higienismo, gobierno de la salud a través de vacunas, aplicaciones móviles, políticas poblacionales de cuarentena, aislamiento y distanciamiento: sus representaciones sociales, conflictos políticos e institucionales. Activismos, ciudadanías e identidades.
- *Dimensión de derechos*: cuestiones de derechos humanos, derecho a la salud, derecho a la información y la comunicación; así como cuestiones de género y sexualidades, desigualdades sociales y económicas, brechas y accesibilidades tecnológicas, discapacidades, diversidades y minorías vulnerables; infancias, juventudes y vejeces.
- *Dimensión epistemológica*: cientificidad, polémicas y validación del conocimiento, desafíos de las prácticas de investigación científicas, adaptación de los procesos de publicación científica, implicancias epistemológicas y comunicacionales del desarrollo de vacunas.
- *Dimensión traumática de la pandemia*: abordaje de diversas formas de malestares, padecimientos, pánicos morales, dolor, duelo, secuelas, padecimientos y aflicciones; manifestaciones y abordajes en salud mental, implicancias subjetivas, sanitarias y políticas.
- *Dimensión geopolítica*: problemáticas vinculadas a fronteras, migraciones, refugiados, conflictos internacionales, decolonialidad y globalización, debates en torno a organismos, y entidades internacionales y multilaterales como la Organización Mundial de la Salud, el plan *Covid-19 vaccines Global Access Facility* (COVAX), etc.
- *Dimensión temporal*: Problematización de las temporalidades de la pandemia, controversias en torno a las expresiones “pospandemia” y “nueva normalidad”, representaciones y reflexiones de sus implicancias. Periodizaciones de la pandemia, comparaciones de momentos, manifestaciones y abordajes.
- *Dimensión espacial*: ambiente, hábitat, urbanismo, adaptación de la infraestructura habitacional, que abarque cuestiones como la ventilación de espacios cerrados, la circulación y uso del espacio público y doméstico.
- *Dimensión artística*: manifestaciones literarias, artísticas y estéticas, incluyendo producción de contenidos digitales, fotográficos, gráficos, audiovisuales en y sobre la pandemia de covid-19.

Los ocho trabajos aceptados, provenientes de Argentina, Brasil, Colombia, EEUU y México, abordan de manera directa y/o ponen en diálogo diversos matices del amplio arco temático propuesto, a través de investigaciones empíricas o abordajes reflexivos sobre la pandemia en distintos contextos nacionales de la región. Los artículos entretejen el análisis comunicacional-informativo, los procesos de digitalización, la perspectiva de derechos, la mirada temporal, espacial y geopolítica, la dimensión traumática de la pandemia y la problematización biopolítica, entre otros planos.

La selección de artículos está integrada por el trabajo de Rosalynn Adeline Vega, titulado “Vacilación ante la vacuna contra el covid-19 en Estados Unidos de América: un estudio etnográfico digital”^1^; el estudio de María de las Nieves Ganiele *et al*., “Alcances y limitaciones de la teleconsulta en pandemia de covid-19: relatos de profesionales de la salud del primer nivel de atención de la Ciudad Autónoma de Buenos Aires”^2^; el análisis de Flavia Demonte *et al*., sobre “Conversación pública sobre vacunas en la pandemia de covid-19 en Argentina, 2021-2022”^3^; el artículo de Luz Adriana Muñoz-Duque y Nidia Elena Ortiz sobre “Las relaciones personas-espacio público: Reflexiones sobre transformaciones, usos normativos, reducciones y contradicciones del espacio público en pandemia”^4^; el aporte de João Vitor Antunes Lins dos Santos *et al*., titulado “Hombres que trabajan a través de aplicaciones móviles en Brasil: reflexiones desde la salud ocupacional”^5^; el trabajo de Paola Consuelo Ladino Marín y Rodolfo Prada Penagos titulado “Análisis del encuadramiento periodístico en tiempos de pandemia de covid-19 en los principales diarios de Colombia: El Tiempo y El Espectador”^6^; el de Doris Elena Muñoz-Zapata y Johanna Marcela Osorio-Franco, sobre “Redes sociales como escenarios para la visibilización de las violencias basadas en género durante la pandemia de covid-19 en Colombia”^7^; y el artículo de Munique Therense y André Luiz Machado das Neves sobre “Redes vivas em salud en la pandemia de covid-19: evaluación del curso de formación multiprofesional ‘Nós da Linha de Frente’ en Manaus, Amazonas, Brasil”^8^.

Queremos resaltar el trabajo de asumir como objeto de interrogación la pandemia de covid-19 como un desafío múltiple que conjuga la temporalidad y condiciones de la pandemia en otra más extensa que llega a nuestro presente. En ese sentido, creemos que la convocatoria y la publicación de los artículos aceptados supone, tanto para autoras y autores como para sus potenciales lectores, un ejercicio de toma de distancia, objetivación y reflexión sobre las propias prácticas que nos retrotrae a las condiciones vividas en pandemia y sus consecuencias, así como también permite pensarlas a la luz del tiempo transcurrido y su proyección. Incluso, consideramos que, para quienes trabajamos diversas problemáticas que articulan lo social, lo comunicacional y la salud, la pandemia nos ha interpelado doblemente, como sujetos sociales atravesados por esa coyuntura y como sujetos epistémicos productores de conocimiento que asumen la responsabilidad de pensar su propio presente. He allí el doble valor de los trabajos reunidos por esta convocatoria.
